# Comparative Nectary Morphology across Cleomaceae (Brassicales)

**DOI:** 10.3390/plants12061263

**Published:** 2023-03-10

**Authors:** Brandi Zenchyzen, Stacie Weissner, Jaymie Martin, Ainsley Lopushinsky, Ida John, Ishnoor Nahal, Jocelyn C. Hall

**Affiliations:** Department of Biological Sciences, University of Alberta, Edmonton, AB T6G 2E9, Canada

**Keywords:** Cleomaceae, evo-devo, floral nectary, floral rewards, floral structure, morphology, nectarostomata, nectar secretion

## Abstract

Floral nectaries have evolved multiple times and rapidly diversified with the adaptive radiation of animal pollinators. As such, floral nectaries exhibit extraordinary variation in location, size, shape, and secretory mechanism. Despite the intricate ties to pollinator interactions, floral nectaries are often overlooked in morphological and developmental studies. As Cleomaceae exhibits substantial floral diversity, our objective was to describe and compare floral nectaries between and within genera. Floral nectary morphology was assessed through scanning electron microscopy and histology across three developmental stages of nine Cleomaceae species including representatives for seven genera. A modified fast green and safranin O staining protocol was used to yield vibrant sections without highly hazardous chemicals. Cleomaceae floral nectaries are most commonly receptacular, located between the perianth and stamens. The floral nectaries are supplied by vasculature, often contain nectary parenchyma, and have nectarostomata. Despite the shared location, components, and secretory mechanism, the floral nectaries display dramatic diversity in size and shape, ranging from adaxial protrusions or concavities to annular disks. Our data reveal substantive lability in form with both adaxial and annular floral nectaries interspersed across Cleomaceae. Floral nectaries contribute to the vast morphological diversity of Cleomaceae flowers and so are valuable for taxonomic descriptions. Though Cleomaceae floral nectaries are often derived from the receptacle and receptacular nectaries are common across flowering plants, the role of the receptacle in floral evolution and diversification is overlooked and warrants further exploration.

## 1. Introduction

Plant–animal interactions have played a crucial role in the rapid diversification of flowering plants [1]. Most flowering plants have evolved a mutualistic relationship with animals in which floral rewards are exchanged for pollen transfer [2,3]. Consequently, flowering plants exhibit an array of morphological features and chemical signals to appeal to the visual and olfactory capabilities and preferences of animal visitors [1,4]. Functioning as a floral reward and in reproduction, pollen has two mutually incompatible purposes and requires resource intensive excess production for animal-mediated pollination [1]. As an alternative, nectar is easier for flowers to produce and animals to metabolize, deterring animals from exclusively consuming reproductively essential pollen [1]. As such, floral nectaries have evolved independently several times throughout flowering plant diversification [4,5]. Despite their prevalence and ecological significance, floral nectaries have been largely overlooked in morphological and systematic studies resulting in outstanding questions regarding their diversity and development as well as evolutionary patterns across flowering plants [4,5].

Although unified by their ability to secrete complex sugary solutions for animal-mediated pollination, floral nectaries exhibit substantial morphological diversity [4,5]. Floral nectaries are diverse in size and shape but can be separated into two forms: structured and well-differentiated from adjacent tissue, or unstructured and inconspicuous but evident by the secretion of nectar [2,4]. Structured floral nectaries typically consist of three components: vasculature that supplies phloem sap, nectary parenchyma that modifies phloem sap or stored starches to produce nectar, and the epidermis that secretes nectar [1,2]. These components may originate from various floral structures; therefore, floral nectaries can be located anywhere in the flower but are often basally situated to ensure visitors contact the reproductive organs while accessing nectar [1,4]. In addition, there are several means of nectar secretion including: secretory trichomes; epidermal cell wall or cuticle rupture; and most commonly, modified stomata (nectarostomata) [1,6]. Floral nectary location, structure, and secretory mechanisms vary substantively across flowering plants and their diversity and evolutionary patterns within families are largely unexplored [4]. Further, the extent to which these structural variations are correlated to family and genera delimitations has been minimally addressed [4].

Cleomaceae is particularly well-suited for comparative developmental investigations. Sister to Brassicaceae, Cleomaceae is a relatively small family of approximately 270 species that houses significant floral diversity [7]. Cleomaceae has a cosmopolitan distribution but is most common in warmer environments such as arid deserts, grasslands, and humid forests [7,8,9]. Cleomaceae flowers vary in symmetry and organ colour, number, size, and elaboration (i.e., gynophores and androgynophores) [7,9]. Much of the morphological variation in Cleomaceae flowers represent understudied components associated with pollinator attraction and rewards [7]. Floral nectaries are one such feature that remains relatively undocumented despite exhibiting diverse morphology across the family. Cleomaceae floral nectaries tend to develop from the receptacle tissue between the perianth and stamens (i.e., extrastaminal), after initiation and considerable growth of the perianth and reproductive structures [10,11]. Though most often located on the receptacle, the floral nectaries can also be derived from petal tissue and can vary in form from annular disks to elaborate adaxial protrusions [12,13,14,15]. The morphologically diverse floral nectaries presumably influence the range in Cleomaceae pollinators. Although there is limited research on Cleomaceae pollination, studies suggest the family primarily consists of generalist species, pollinated by a variety of insects such as bees, flies, and butterflies [16,17,18]. However, some species (*Melidiscus giganteus* and *Tarenaya houtteana*) may be specialists, exclusively pollinated by bats [19,20]. Regardless of the pollination syndrome, nectar plays a vital role in rewarding the array of pollinators [16,17,18,19,20]. Yet, Cleomaceae floral nectaries are scarcely mentioned in species descriptions [21] and their architecture and ultrastructure have not been characterized in detail across the family.

**Figure 1:**
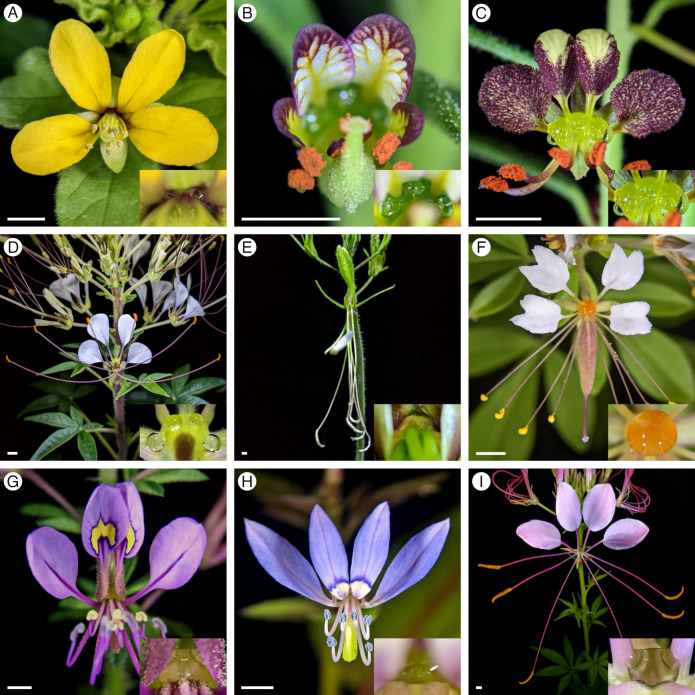
Anthetic flower with nectary inset of the nine Cleomaceae species. (**A**) *Arivela viscosa*. (**B**) *Cleome amblyocarpa*. (**C**) *Cleome violacea*. (**D**) *Gynandropsis gynandra*. (**E**) *Melidiscus giganteus*. (**F**) *Polanisia dodecandra*. (**G**) *Sieruela hirta*. (**H**) *Sieruela rutidosperma*. (**I**) *Tarenaya houtteana*. The scale bar represents 0.25 cm in all images to illustrate the drastic variation in flower size.

This work represents the first detailed comparative morphological investigation of floral nectaries across Cleomaceae and within genera, complementing brief comparisons of floral nectaries [12,13,14] and more comprehensive developmental studies on floral symmetry and stamen number [10,11]. We studied nine species (Figure 1) including representatives scattered across seven of the 13 major clades in Cleomaceae [22]. For two of the clades (*Cleome* L. and *Sieruela* Raf.), we selected two species for within-genera comparisons. In addition to the phylogenetic distribution, this sampling of species reflects some of the floral diversity in Cleomaceae with taxa exhibiting a range of flower size, colour, and organ number and elaboration (Figure 1). We examined floral nectaries using visual observations, scanning electron microscopy, and a modified histological approach to (1) describe floral nectary position, structure, and internal anatomy; (2) characterize the mode of nectar secretion; and (3) evaluate patterns of floral nectary traits across and within genera.

## 2. Results

### 2.1. Arivela viscosa

**Figure 2:**
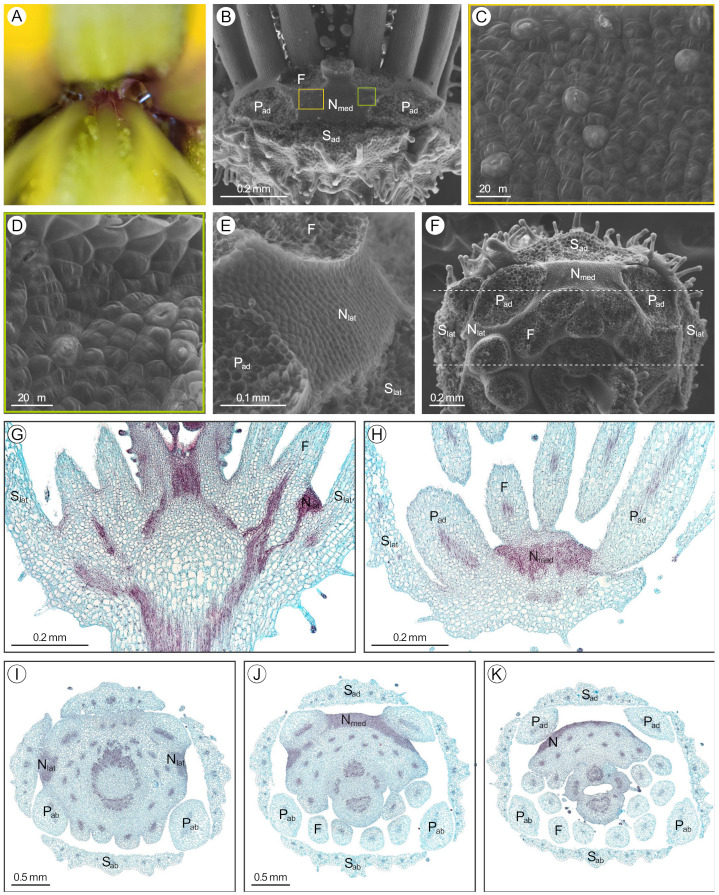
*Arivela viscosa* nectary (**A**) photograph, (**B**–**F**) scanning electron micrographs, and (**G**–**K**) fast green and safranin O-stained sections. (**A**) Apical view of the nectary. (**B**) Adaxial view of the nectary. (**C**–**E**) Adaxial view close-ups: (**C**,**D**) medial nectary lobe, corresponding to the left and right boxes in (**B**), respectively, and (**E**) lateral nectary lobe. (**F**) Apical view of the nectary. (**G**,**H**) Longitudinal sections (frontal plane), corresponding to the bottom and top dashed lines in (**F**), respectively. (**I**–**K**) Transverse sections of the nectary from proximal to distal positioning. All images are of anthetic stage specimens. F: filament; N_lat_: lateral nectary lobe; N_med_: medial nectary lobe; P_ab_: abaxial petal; P_ad_: adaxial petal; S_ab_: abaxial sepal; S_ad_: adaxial sepal; S_lat_: lateral sepal.

*Arivela viscosa* (L.) Raf. has an inconspicuous adaxial extrastaminal nectary detectable by a small volume of nectar at the base of the adaxial petals and stamen filaments (hereafter referred to as filaments; Figure 2A). The nectary has three lobes, a medial lobe connected to two lateral lobes by narrow stretches of nectariferous tissue between the adaxial petals and filaments (Figure 2B,F). The nectary lobes are slightly convex while the base of the adaxial petals and filaments form a concavity for the narrow stretches of nectariferous tissue. Throughout development, the three nectary lobes increase in size. In the bud stage, the green nectary is challenging to distinguish from the surrounding green tissue. However, as the flower develops, maroon pigment accumulates at the base of the sepals, petals, and filaments, making the green nectary marginally less discreet (Figure 2A). Nectarostomata are primarily found on the medial lobe and the narrow stretches of nectariferous tissue, with few located on the lateral lobes (Figure 2C–E). In addition, nectarostomata are mainly situated on the distal half of the nectary, closer to the filaments. Small amounts of granular material can be found exuding from the nectarostomata openings (Figure 2C,D). Nectary parenchyma (red-stained tissue) is present at the medial lobe, the concavities between the adaxial petals and filaments, and the lateral lobes, but does not occupy a large portion of the receptacle (Figure 2G–K). Vasculature diverges to supply the nectary and adjacent perianth and stamens (Figure 2G).

### 2.2. Cleome amblyocarpa

**Figure 3:**
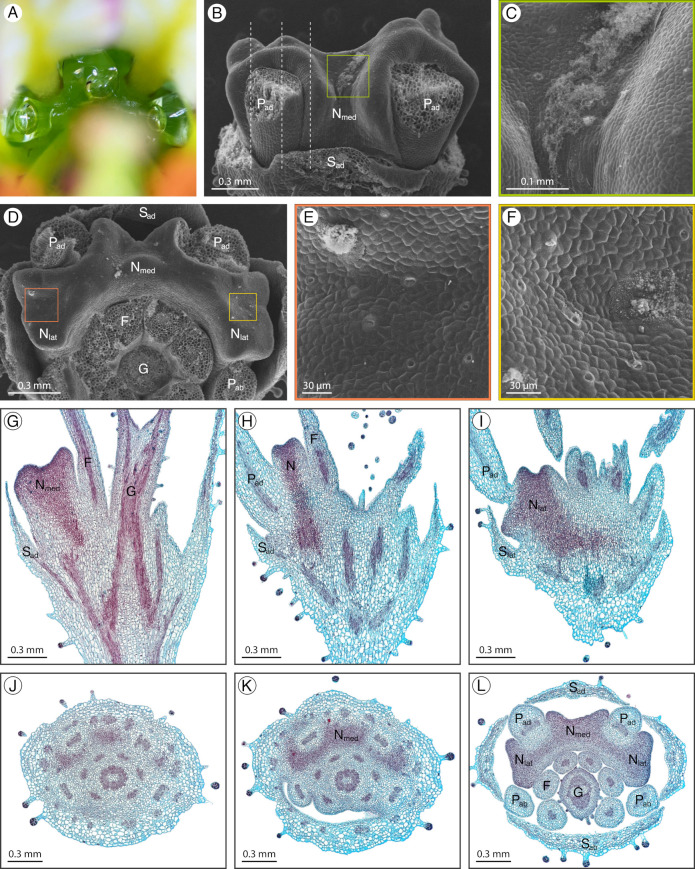
*Cleome amblyocarpa* nectary (**A**) photograph, (**B**–**F**) scanning electron micrographs, and (**G**–**L**) fast green and safranin O-stained sections. (**A**) Apical view of the nectary. (**B**) Adaxial view of the nectary. (**C**) Adaxial view close-up of the medial nectary lobe, corresponding to the box in (**B**). (**D**) Apical view of the nectary. (**E**,**F**) Apical view close-ups of the lateral nectary lobes, corresponding to the left and right boxes in (**D**), respectively. (**G**–**I**) Longitudinal sections (sagittal plane) of the nectary, corresponding to the dashed lines in (**B**) from right to left, respectively. (**J**–**L**) Transverse sections of the intermediate stage nectary from proximal to distal positioning. All images are of anthetic stage specimens unless indicated otherwise. F: filament; G: gynophore; N_lat_: lateral nectary lobe; N_med_: medial nectary lobe; P_ab_: abaxial petal; P_ad_: adaxial petal; S_ab_: abaxial sepal; S_ad_: adaxial sepal.

*Cleome amblyocarpa* Barratte and Murb. has a structured adaxial extrastaminal nectary with a complex form protruding from the receptacle (Figure 3A). The nectary has convex rims wrapping around the adaxial petals and filaments with concavities between these rims (Figure 3B,D). There are three nectary lobes, the medial lobe with an adaxial concavity between the adaxial petals, and two lateral lobes with apical concavities. During development, the nectary remains green while the convex rims and concavities become more pronounced. Nectarostomata are primarily scattered throughout the adaxial concavity of the medial lobe and the apical concavities of the lateral lobes and are often associated with the granular substance (Figure 3C,E,F). Nectar secretion corresponds to the location of nectarostomata. Nectary parenchyma is present at the apex of the nectary and spans down to the level of sepal attachment but is mainly absent from nectary tissue adjacent to the filaments (Figure 3G–L). Vasculature diverges from the perianth supply to feed the nectary (Figure 3G,I).

### 2.3. Cleome violacea

**Figure 4:**
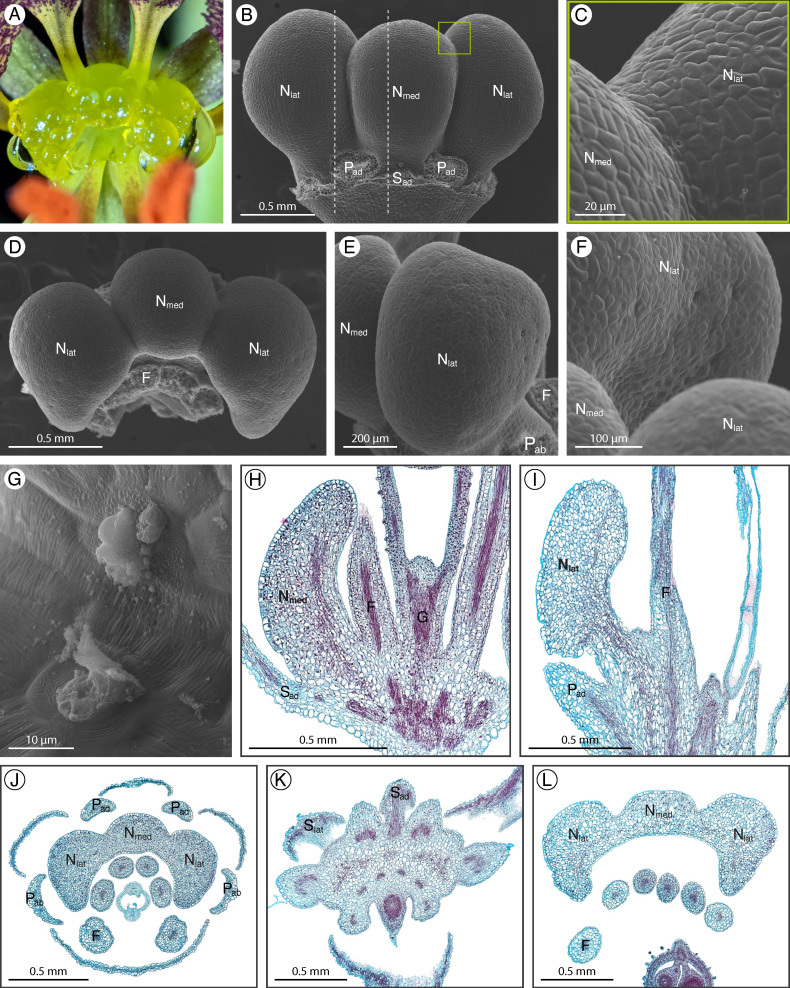
*Cleome violacea* nectary (**A**) photograph, (**B**–**G**) scanning electron micrographs, and (**H**–**L**) fast green and safranin O-stained sections. (**A**) Apical view of the nectary. (**B**) Adaxial view of the nectary. (**C**) Adaxial view close-up of the medial and lateral nectary lobes, corresponding to the box in (**B**). (**D**) Apical view of the nectary. (**E**) Lateral view of the nectary. (**F**) Lateral view close-up of the nectary. (**G**) Close-up of nectarostomata. (**H**,**I**) Longitudinal sections (sagittal plane) of the nectary, corresponding to the right and left dashed lines in (**B**), respectively: (**H**) intermediate stage nectary, and (**I**) anthetic stage nectary. (**J**–**L**) Transverse sections: (**J**) intermediate stage nectary, (**K**,**L**) anthetic stage nectary from proximal to distal positioning. All images are of anthetic stage specimens unless indicated otherwise. F: filament; G: gynophore; N_lat_: lateral nectary lobe; N_med_: medial nectary lobe; P_ab_: abaxial petal; P_ad_: adaxial petal; S_ad_: adaxial sepal; S_lat_: lateral sepal.

*Cleome violacea* L. has a structured adaxial extrastaminal nectary protruding from the receptacle (Figure 4A). The nectary has three prominent convex lobes, one medial lobe, and two lateral lobes (Figure 4B,D). From bud to flower, the nectary lobes become larger and more pronounced but remain green. Nectar droplets form on the apical, lateral, and abaxial surfaces of the nectary, corresponding to the location of nectarostomata. Nectarostomata are scattered about the apical and lateral surfaces (Figure 4C,E), including the apical crevices between lobes (Figure 4G), and are also positioned on the abaxial surface of the nectary, adjacent to the stamens (Figure 4F). The nectarostomata are often slightly sunken amongst the epidermal cells. The granular material can be found in nectarostomata openings (Figure 4G). Unlike the other eight species examined here, the nectary of *C. violacea* lacks prominent red-stained parenchyma. The nectary of pre-anthetic flowers tends to contain cells that are slightly stained red (Figure 4H) but this is not always the case (Figure 4J). Instead, the nectary contains vasculature which diverges from the perianth supply (Figure 4H) and extends from the receptacle to the apex of the nectary, along the abaxial half of the nectary lobes (Figure 4I,K,L).

### 2.4. Gynandropsis gynandra

**Figure 5:**
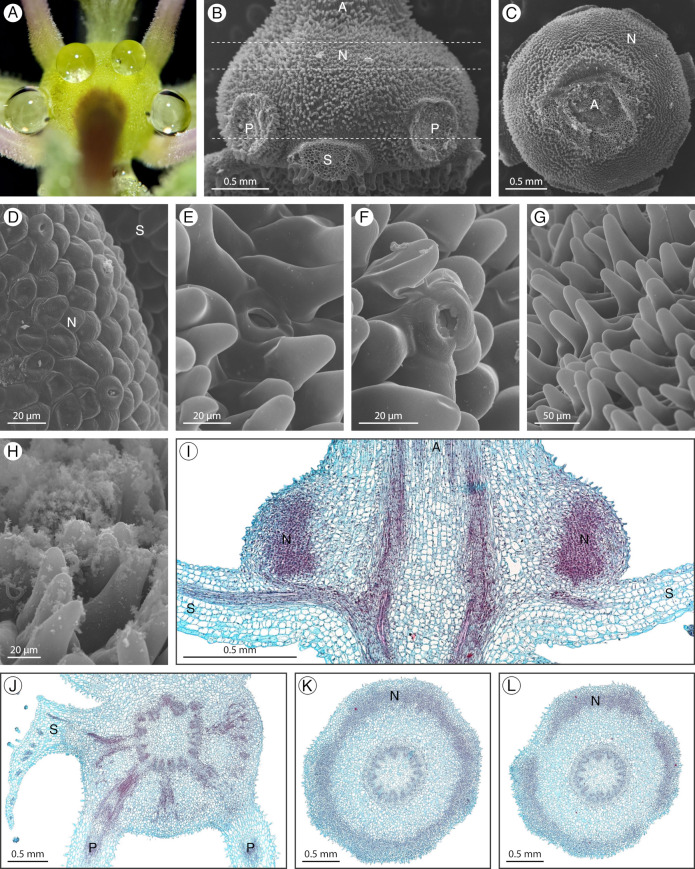
*Gynandropsis gynandra* nectary (**A**) photograph, (**B**–**H**) scanning electron micrographs, and (**I**–**L**) fast green/safranin O-stained sections. (**A**) Apical view of the nectary. (**B**) Side view of the nectary. (**C**) Apical view of the nectary. (**D**) Apical view close-up of the bud stage nectary. (**E**,**F**) Close-up of nectarostomata. (**G**,**H**) Close-up of the nectary. (**I**) Longitudinal section of the intermediate stage nectary. (**J**–**L**) Transverse sections of the nectary, corresponding to the dashed lines in (**B**) from proximal to distal, respectively. All images are of anthetic stage specimens unless indicated otherwise. A: androgynophore; F: filament; N: nectary; P: petal; S: sepal.

*Gynandropsis gynandra* (L.) Briq. has an inconspicuous annular extrastaminal nectary detectable by the presence of 4–5 nectar droplets (Figure 5A). One nectar droplet is secreted opposite the four sepals, or rather than one adaxial nectar droplet, two nectar droplets are formed opposite the adaxial petals. During development, the nectary increases in size and transitions from a darker green to a lighter green. Occasionally, purple pigmentation accumulates at the sites of nectar secretion. The nectary is a convex ring covered in distinctive cells with finger-like projections (Figure 5B,C,G). Nectarostomata are primarily positioned at the base of these cells (Figure 5E) and are rarely found at their apex (Figure 5F). Due to the protruding cells, nectarostomata can be difficult to find but are easier to locate in the bud stage before the cellular extensions have developed (Figure 5D). Nectarostomata along with the granular residue are mainly located on the apical half of the nectary, opposite the sepals but can also be found opposite the petals (Figure 5B,D,H). Throughout most of the nectary, the nectary parenchyma is annular, forming a ring near the epidermis (Figure 5K). However, near the apex of the nectary, the nectary parenchyma is divided into four regions (Figure 5L). These four regions of nectary parenchyma are opposite the sepals and correspond to the positions of the four nectar droplets. The nectary is supplied by vasculature diverging from the perianth supply (Figure 5I,J).

### 2.5. Melidiscus giganteus

**Figure 6:**
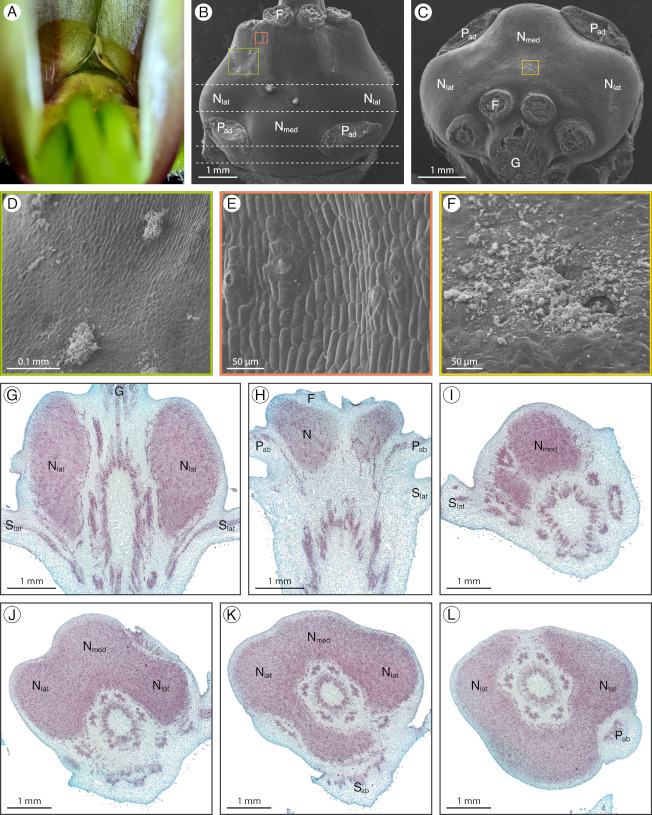
*Melidiscus giganteus* nectary (**A**) photograph, (**B**–**F**) scanning electron micrographs, and (**G**–**L**) fast green and safranin O-stained sections. (**A**) Apical view of the nectary. (**B**) Adaxial view of the nectary. (**C**) Apical view of the nectary. (**D**,**E**) Adaxial view close-ups, corresponding to the bottom and top boxes in (**B**), respectively. (**F**) Apical view close-up, corresponding to the box in (**C**). (**G**,**H**) Longitudinal sections (frontal plane): (**G**) lateral nectary lobes and (**H**) abaxial side of nectary. (**I**–**L**) Transverse sections of the nectary, corresponding to the dashed lines in (**B**) from proximal to distal, respectively. All images are of anthetic stage specimens. F: filament; G: gynophore; N_lat_: lateral nectary lobe; N_med_: medial nectary lobe; P_ab_: abaxial petal; P_ad_: adaxial petal; S_ab_: abaxial sepal; S_ab_: abaxial sepal; S_lat_: lateral sepal.

*Melidiscus giganteus* (L.) Raf. has a large and structured annular extrastaminal nectary (Figure 6A). The nectary has three main convex lobes, one medial lobe between the adaxial petals, and two lateral lobes between the adaxial and abaxial petals (Figure 6B,C). The distal half of the nectary is narrower than the proximal half. Typically, the abaxial side of the nectary does not have a prominent lobe. Most of the nectary remains light green throughout development. However, maroon pigment accumulates at the base of the nectary, above the sepal and petal bases. Nectar is secreted at the adaxial surface of the nectary and held in place by the base of the petals. Nectarostomata and clusters of the granular substance are primarily located on the distal half of the nectary, exclusively on the adaxial side (Figure 6D–F). The nectary parenchyma occupies a large volume, extending from the nectary apex to the level of sepal attachment but is absent from the nectary tissue immediately adjacent to the epidermis (Figure 6G,I). Although nectar is only secreted on the adaxial surface of the nectary, the nectary parenchyma is annular, wrapping closely around the vasculature leading to the stamens (Figure 6J–L). Vasculature diverges from the perianth supply and is visible within the nectary parenchyma (Figure 6H) and along the inner boundary of nectary parenchyma near the staminal vascular supply (Figure 6G). The nectary varies substantially as the plant ages, becoming smaller with less defined lobes and fewer nectarostomata. In addition, nectary parenchyma is only found in the lateral nectary lobes and nectar production tends to cease in growth chamber conditions.

### 2.6. Polanisia dodecandra

**Figure 7:**
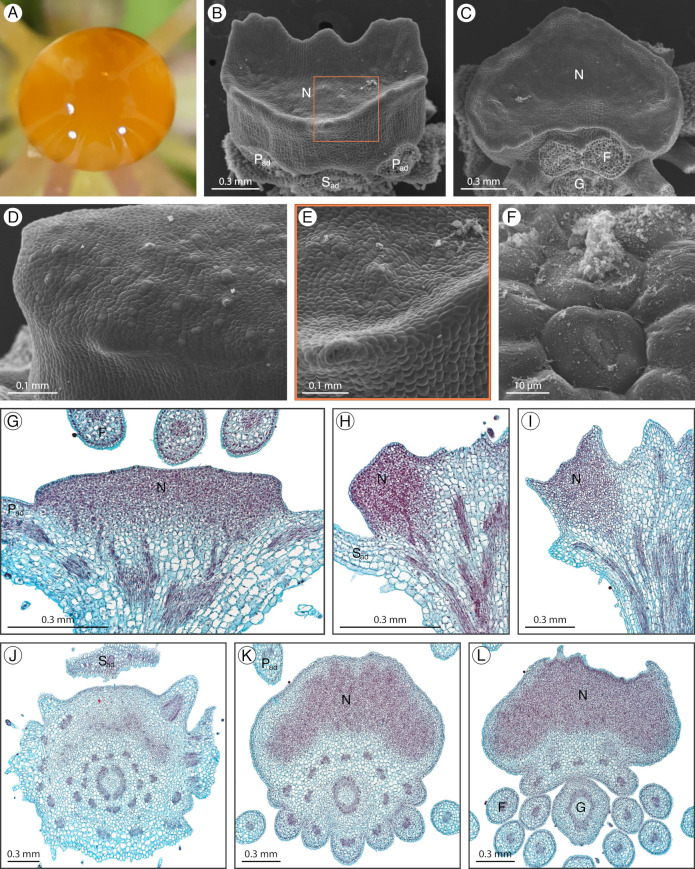
*Polanisia dodecandra* nectary (**A**) photograph, (**B**–**F**) scanning electron micrographs, and (**G**–**L**) fast green and safranin O-stained sections. (**A**) Apical view of the nectary. (**B**) Adaxial view of the nectary. (**C**) Apical view of the nectary. (**D**–**F**) Adaxial view close-ups: (**D**) bud stage nectary, (**E**) anthetic stage nectary, corresponding to the box in (**B**), and (**F**) anthetic stage nectarostomata. (**G**) Longitudinal section (frontal plane) of the intermediate stage nectary. (**H**,**I**) Longitudinal section (sagittal plane): (**H**) intermediate stage nectary and (**I**) anthetic stage nectary. (**J**–**L**) Transverse sections of the nectary from proximal to distal positioning. All images are of anthetic stage specimens unless indicated otherwise. F: filament; G: gynophore; N: nectary; P_ad_: adaxial petal; S_ad_: adaxial sepal.

*Polanisia dodecandra* (L.) DC. has a structured adaxial extrastaminal nectary protruding from the receptacle (Figure 7A). The nectary has a somewhat cordate-shaped concavity at its apex (Figure 7B–C) where nectar is secreted and held. The nectary is faintly coloured with purple pigment in the bud stage; however, as the flower develops, vibrant orange pigment accumulates. The apical surface of the nectary is relatively flat at the bud and intermediate stages (Figure 7D,G–H), but has an encompassing lip creating a cup-shape at the anthetic stage (Figure 7B,I). Nectarostomata are exclusively located on the apical surface of the nectary (Figure 7D–F). The granular deposit is often found in the apical concavity, near nectarostomata (Figure 7E,F). Nectary parenchyma is present throughout much of the nectary, excluding the exterior edges of the anthetic stage nectary (Figure 7K-L), and extending to the level of sepal attachment (Figure 7J). The nectary is supplied by vasculature which diverges from the perianth supply (Figure 7G–I), with vasculature sometimes visible within the nectary parenchyma (Figure 7H).

### 2.7. Sieruela hirta

**Figure 8:**
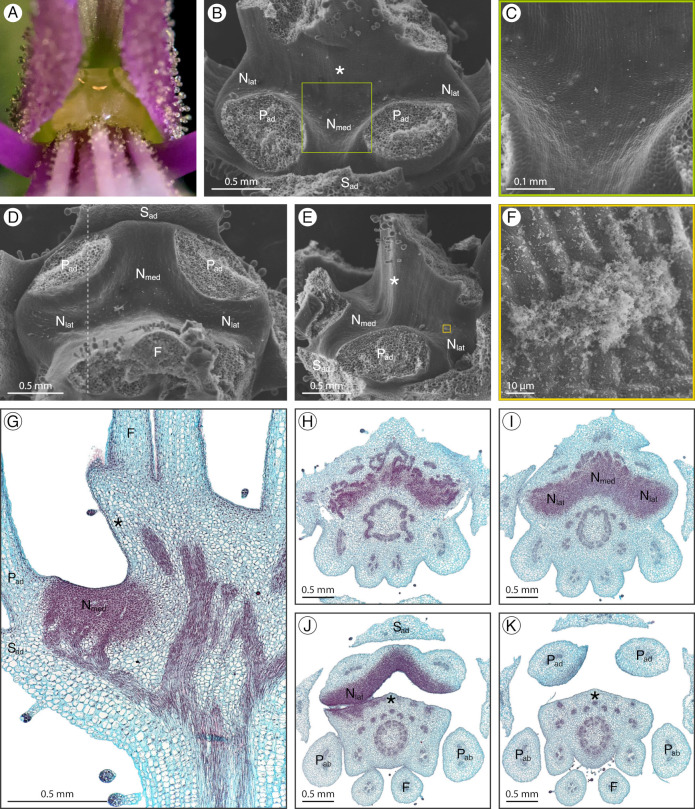
*Sieruela hirta* nectary (**A**) photograph, (**B**–**F**) scanning electron micrographs, and (**G**–**K**) fast green and safranin O-stained sections. (**A**) Apical view of the nectary. (**B**) Adaxial view of the nectary. (**C**) Adaxial view close-up of the nectary, corresponding to the box in (**B**). (**D**) Apical view of the nectary. (**E**) Lateral view of the nectary. (**F**) Lateral view close-up of the nectary, corresponding to the box in (**E**). (**G**) Longitudinal section (sagittal plane) of the intermediate stage nectary, corresponding to the dashed line in (**D**). (**H**–**K**) Transverse sections of the nectary from proximal to distal orientation. All images are of anthetic stage specimens unless indicated otherwise. Basal fusion of adaxial filaments is indicated with an asterisk. F: filament; N_lat_: lateral nectary lobe; N_med_: medial nectary lobe; P_ab_: abaxial petal; P_ad_: adaxial petal; S_ad_: adaxial sepal.

*Sieruela hirta* (Klotzsch) Roalson and J. C. Hall has a structured adaxial extrastaminal nectary depressing into the receptacle (Figure 8A). The concavity spans from the lateral nectary lobes between the adaxial petals and filaments, to the medial nectary lobe between the adaxial petals (Figure 8B,D). The adaxial filaments are basally fused, forming a wall along the nectary (Figure 8E). Nectar is held within the nectary concavity and basally fused adaxial filaments. During development, the nectary concavity becomes more pronounced and the adaxial filament wall extends while the nectary remains light green. Nectarostomata are congregated within the nectary concavity and are absent from the adaxial filament wall (Figure 8C). The granular substance is found along with the nectarostomata in the nectary concavity (Figure 8F). Nectary parenchyma is present in the nectary concavity and spans to the level of sepal attachment but is absent from the adaxial filament wall (Figure 8G–K). The vasculature diverges from the strands leading to the perianth to supply the nectary (Figure 8G).

### 2.8. Sieruela rutidosperma

**Figure 9:**
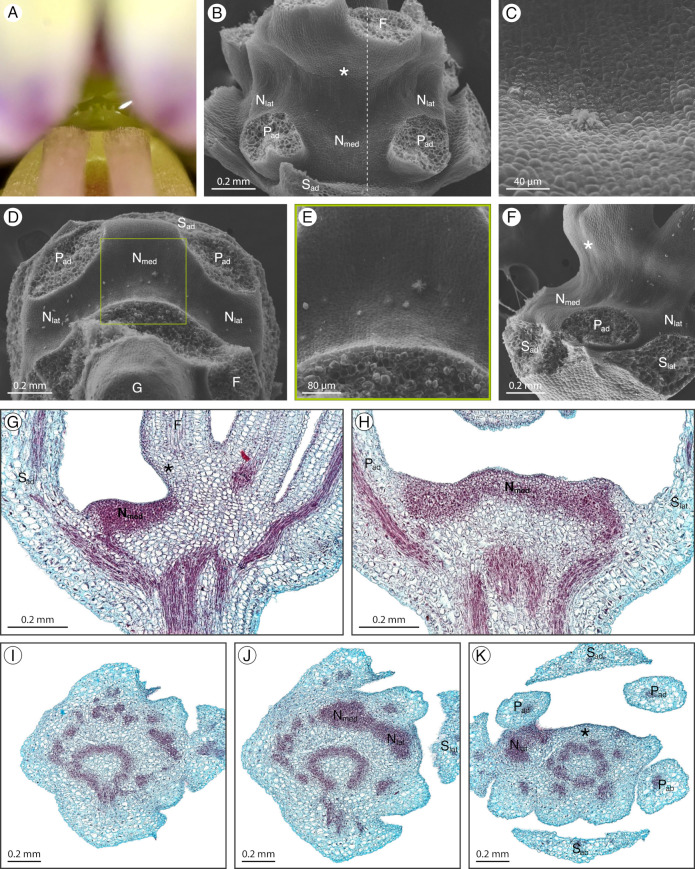
*Sieruela rutidosperma* nectary (**A**) photograph, (**B**–**F**) scanning electron micrographs, and (**G**–**K**) fast green and safranin O-stained sections. (**A**) Apical view of the nectary. (**B**) Adaxial view of the nectary. (**C**) Adaxial view close-up of the medial nectary lobe. (**D**) Apical view of the nectary. (**E**) Apical view close-up of the nectary, corresponding to the box in (**D**). (**F**) Lateral view of the nectary. (**G**) Longitudinal section (sagittal plane) of the intermediate stage nectary, corresponding to the dashed line in (**B**). (**H**) Longitudinal section (frontal plane) of the nectary. (**I**–**K**) Transverse section of the nectary from proximal to distal positioning. All images are of anthetic stage specimens unless indicated otherwise. Basal fusion of adaxial filaments is indicated with an asterisk. F: filament; G: gynophore; N_lat_: lateral nectary lobe; N_med_: medial nectary lobe; P_ab_: abaxial petal; P_ad_: adaxial petal; S_ab_: abaxial sepal; S_ad_: adaxial sepal; S_lat_: lateral sepal.

*Sieruela rutidosperma* (DC.) Roalson and J. C. Hall has a structured adaxial extrastaminal nectary depressing into the receptacle (Figure 9A). The concavity extends from the lateral nectary lobes between the adaxial petals and filaments, to the medial nectary lobe between the adaxial petals (Figure 9B,D). The adaxial filaments are basally fused, forming a wall curved toward the adaxial sepal (Figure 9F). Nectar accumulates between the nectary concavity and the curved adaxial filament wall. Throughout development, the nectary concavity becomes more distinct and the adaxial filament wall extends, increasing in curvature. The nectary remains light green from bud to anthesis. Nectarostomata along with the granular deposit are located within the nectary concavity (Figure 9C,E). Nectarostomata are absent from the basally fused adaxial filaments. Nectary parenchyma is present within the nectary concavity but is shallow and does not occupy the adaxial filament wall (Figure 9G–K). The nectary is supplied by vasculature which diverges from the perianth supply (Figure 9G,H).

### 2.9. Tarenaya houtteana

**Figure 10:**
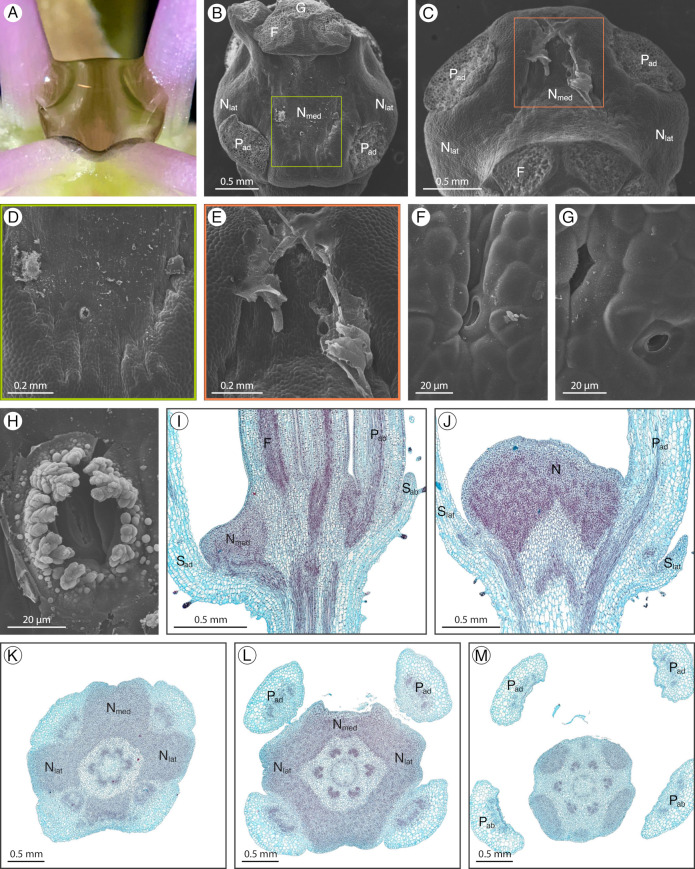
*Tarenaya houtteana* nectary (**A**) photograph, (**B**–**H**) scanning electron micrographs, and (**I**–**M**) fast green and safranin O-stained sections. (**A**) Apical view of the nectary. (**B**) Adaxial view of the nectary. (**C**) Apical view of the nectary. (**D**) Adaxial view close-up of the nectary, corresponding to the box in (**B**). (**E**) Apical view close-up of the nectary, corresponding to the box in (**C**). (**F**–**H**) Close-up of nectarostomata. (**I**) Longitudinal section (sagittal plane) of the bud stage nectary. (**J**) Longitudinal section (frontal-oblique plane) of the intermediate stage nectary. (**K**–**M**) Transverse sections of the nectary from proximal to distal positioning. All images are of anthetic stage specimens unless indicated otherwise. F: filament; G: gynophore; N_lat_: lateral nectary lobe; N_med_: medial nectary lobe; P_ab_: abaxial petal; P_ad_: adaxial petal; S_ab_: abaxial sepal; S_ad_: adaxial sepal; S_lat_: lateral sepal.

*Tarenaya houtteana* (Chodat) Iltis (formerly *T. hassleriana*; see Neto et al. (2022) for recent taxonomic revision [23]) has a structured extrastaminal annular nectary (Figure 10A). Nectar is secreted on the adaxial surface of the nectary and is held in place by the base of the petals. The nectary has three prominent lobes at its proximal half: a medial lobe between the adaxial petals, and two lateral lobes between the adaxial and abaxial petals (Figure 10B,C). The abaxial side of the flower does not have a well-defined nectary lobe. The distal half of the nectary is narrower than the proximal half. The medial nectary lobe has convex rims wrapping around the adaxial petals, roughly forming a ‘V’ shaped depression pointed toward the adaxial sepal (Figure 10D,E). Wrinkles and folds are present along the ‘V’ shape (Figure 10B,D). The lateral nectary lobes are convex between the adaxial and abaxial petals but have a concave region near the base of the filaments (Figure 10B). The convex rims, wrinkles, and folds of the medial nectary lobe are absent in the bud stage but become apparent in the intermediate stage. Additionally, the nectary increases in size and remains light green during development. Nectarostomata are located on the medial lobe frequently within crevasses (Figure 10F–H). A large amount of the granular substance can often be found covering the wrinkles and folds of the medial nectary lobe (Figure 10E). The nectary parenchyma occupies a large volume, extending to the level of sepal attachment (Figure I,J). Although nectar is only secreted on the adaxial surface of the nectary, nectary parenchyma is found on all sides of the nectary. At the base of the nectary, the nectary parenchyma is present between the petal vasculature (Figure 10K). The nectary parenchyma is annular near the base of the petals, wrapping around the reproductive organ vascular supply (Figure 10L). At the distal half of the nectary, the nectary parenchyma separates into four main regions aligned with the four petals (Figure 10M). The nectary is fed by vasculature which diverges from the perianth supply (Figure 10I,J). For all nine species, the majority of nectarostomata are open at anthesis but can be found closed earlier in development, most often in the bud stage.

## 3. Discussion

**Figure 11:**
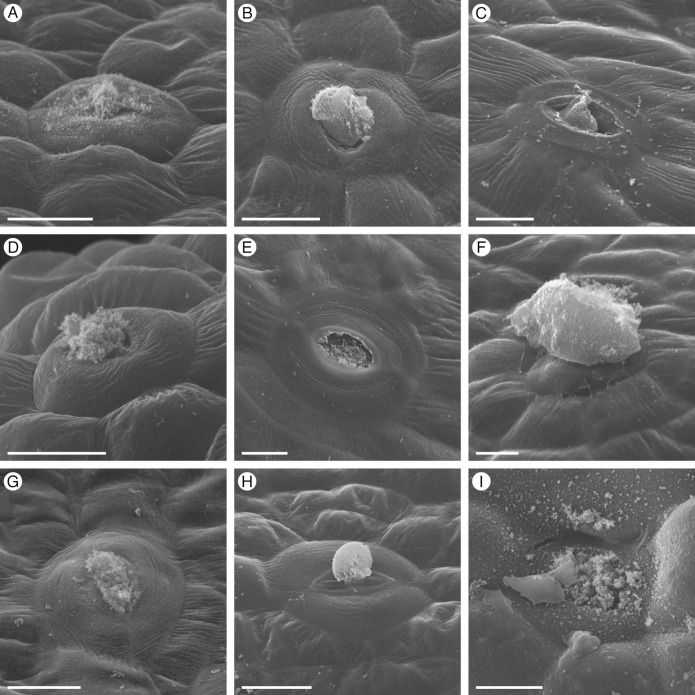
Nectarostomata of the nine Cleomaceae species. (**A**) *Arivela viscosa*. (**B**) *Cleome amblyocarpa*. (**C**) *Cleome violacea*. (**D**) *Gynandropsis gynandra*. (**E**) *Melidiscus giganteus*. (**F**) *Polanisia dodecandra*. (**G**) *Sieruela hirta*. (**H**) *Sieruela rutidosperma*. (**I**) *Tarenaya houtteana*. All images are of anthetic stage specimens, except *C*. *amblyocarpa* (intermediate stage) and *G*. *gynandra* (bud stage). Scale bar represents 10 μm.

Thorough analyses of the nine species revealed striking patterns in floral nectary morphology and anatomy across Cleomaceae (Figure 1). Each species has a nectary located either between the perianth and stamens or perianth and androgynophore; such is the case for *G. gynandra* which has a particularly prominent androgynophore elevating the reproductive organs (Figure 5). The nectaries range from structured protrusions or concavities to inconspicuous and challenging to discern from the receptacle. Nectar is secreted on the adaxial surface of the nectary, apart from *G. gynandra* which secretes one of four to five nectar droplets on the abaxial surface. Excluding the anthetic flowers of *C. violacea*, the nectaries contain nectary parenchyma, highly conspicuous bright red tissue in the fast green and safranin O-stained sections. The volume of nectary parenchyma varies from a small portion in *A. viscosa* (Figure 2), to a large quantity in *M. giganteus* (Figure 6). Corresponding with the site of nectar secretion, the nectary parenchyma is primarily adaxially situated for *A. viscosa*, *C. amblyocarpa*, *C. violacea*, *P. dodecandra*, *S. hirta*, and *S. rutidosperma*; however, the nectary parenchyma is annular forming a ring around the vasculature supplying the reproductive organs for *G. gynandra*, *M. giganteus*, and *T. houtteana*. Regardless of the absence or presence and extent of nectary parenchyma, the nectary of each species is supplied by vasculature which diverges from the vasculature leading to the perianth. Scattered across the nectary epidermis of each species are nectarostomata (Figure 11). Nectarostomata may be closed or opened, most frequently in the bud and flowers stages, respectively. Though the abundance of nectarostomata varies from species to species, a granular substance is located near or extruding from nectarostomata in scanning electron micrographs.

### 3.1. Nectarostomata and Vasculature Are Unifying Features of Cleomaceae Floral Nectaries

Cleomaceae floral nectaries are united by nectarostomata as the mechanism for nectar secretion (Table 1). Nectarostomata are the most common secretory mechanism and have been extensively reported in eudicots and described in some Orchidaceae species [2,4]. Erbar and Leins (1996) previously referred to the nectarostomata of *C. violacea* as nectar slits and, aside from the nine species in our study, nectarostomata have also been noted in *Cleomella sparsifolia* (Cleomaceae) [10,24]. Nectarostomata are often described as continuously open and unable to control nectar secretion [4]. Yet, nectarostomatal aperture regulation has only been thoroughly studied in *Vicia faba* (Fabaceae) [2,25,26]. In this taxon, nectarostomata development is asynchronous with most opening a few days prior to anthesis and rarely closing once mature [25]. Consistent with Davis and Gunning (1992) [25], we observed closed nectarostomata primarily in the bud stage when they are more likely to be immature. However, the possibility that these nectarostomata open later in development to initiate nectar secretion warrants further exploration.

A granular substance was found extruding from nectarostomata and spread across the nectary epidermis for all nine species. This substance has been previously described as “secretory material”, “spongy secretion”, or “granular structures” [6,27,28,29,30] and can often be observed in the scanning electron micrographs of nectary studies, even if not mentioned in text [31,32,33]. It has been hypothesized that the occluding material could be crystallized nectar that may function as an alternative to guard cell movements to close the nectarostomata and perhaps prevent the entry of pathogens [4,25,34]. However, dissolved sugars in the nectar should be washed away during the fixation and dehydration processes and any crystalized sugars in the minute volume of nectar are likely to dissolve in FAA [35]. Further, the granular material is distinct from the waxy cuticle and cellular debris, as observed by Davis and Gunning (1992) [25]. Alternatively, we propose the granular secretion is a remnant of the microbial community inhabiting the nectar. In congruence with our hypothesis, Carey et al. (2023) reported hits to bacteria and yeast-related rRNA in the *Cleome violacea* floral nectary transcriptome and material that looks like budding yeast cells in the nectarostomata openings [36]. Bacteria and fungi, primarily yeast, reside in nectar and can alter its chemical composition, influencing pollinator attraction [4,5]. The relationship between microbial communities in nectar and pollinator interactions is in the early stages of exploration with many intriguing questions remaining [2,5]. Further research is needed to confirm the identity of the granular secretion.

In addition to nectarostomata, the floral nectaries of all nine Cleomaceae species are supplied by vasculature which diverges from the perianth vascular bundles (Table 1). Similar vasculature branching has been reported in *Cleomella serrulata* (Cleomaceae) [12]. Though challenging to observe with the densely stained nectary parenchyma of our specimens, Stoudt (1941) described the vasculature of Cleomaceae floral nectaries as unlignified and profusely branching in the base of the nectary parenchyma and suggested the nectary vascular supply was derived from a former staminal supply [12]. Although the branching patterns and sources vary, vasculature is a shared feature of floral nectaries across angiosperms [4,37]. Whether an evolutionary consequence of removing the excess sugars from phloem or hydrostatic pressure and weak expanding tissue causing “leaky phloem”, floral nectaries shifted from a physiological to an ecological function of secreting the main floral reward [1,4,38].

**Table 1:** Summary of floral nectary characteristics and pollination system for the nine Cleomaceae species.

Species	Nectary Type	Nectary Location	Nectary Parenchyma	Nectary Vasculature	Nectar Secretion Mechanism	Pollination System
*Arivela* *viscosa*	Slightly concave adaxial	Between perianth and stamens	Present	Present	Nectarostomata	Generalist [18,39,40]
*Cleome* *amblyocarpa*	Protruding adaxial	Between perianth and stamens	Present	Present	Nectarostomata	Unknown
*Cleome* *violacea*	Protruding adaxial	Between perianth and stamens	Absent/not prominent	Present	Nectarostomata	Unknown
*Gynandropsis* *gynandra*	Annular	Between perianth and androgynophore	Present	Present	Nectarostomata	Generalist/hawkmoth [18,39,41,42]
*Melidiscus* *giganteus*	Annular	Between perianth and stamens	Present	Present	Nectarostomata	Bat [20]
*Polanisia* *dodecandra*	Protruding adaxial	Between perianth and stamens	Present	Present	Nectarostomata	Generalist [17]
*Sieruela* *hirta*	Concave adaxial	Between perianth and stamens	Present	Present	Nectarostomata	Unknown
*Sieruela* *rutidosperma*	Concave adaxial	Between perianth and stamens	Present	Present	Nectarostomata	Generalist [43]
*Tarenaya* *houtteana*	Annular	Between perianth and stamens	Present	Present	Nectarostomata	Bat [19]

### 3.2. Nectary Parenchyma Is Variable throughout Cleomaceae

Nectary parenchyma varies substantially between Cleomaceae species, from presumably absent in *Cleome violacea* to annular and occupying a large volume in *M. giganteus* (Table 1). Secretion and accumulation of nectar on the adaxial side of the flower tends to correspond with adaxially positioned nectary parenchyma. Yet in *M. giganteus* and *T. houtteana*, nectary parenchyma is annular despite secretion of nectar exclusively on the adaxial surface. Abundance of nectary parenchyma may be one factor positively correlated to nectar volume [4,37]. The volume of nectar secreted is related to pollinator type, a balance between fulfilling the energy needs of the pollinator while encouraging visitation of other flowers [1]. For example, flowers with high energy requirement pollinators, such as hawkmoths and bats, produce more nectar than those with lower energy requirement pollinators, including bees and butterflies [44]. Though some bees and wasps are endothermic (i.e., internally generate heat to regulate body temperature) and thus have higher energy needs, pollinators with larger body sizes such as hawkmoths and bats require more energy per individual [1,45]. The three species from our study that have annular nectary parenchyma also have high energy requirement pollinators (*G. gynandra*, *M. giganteus*, and *T. houtteana*; Table 1). Although nectar secretion exclusively occurs on the adaxial surface of the nectary for *M. giganteus* and *T. houtteana*, the extensive annular nectary parenchyma may allow the flower to produce enough nectar for bats. *Gynandropsis gynandra* is unique in that nectar secretion is not restricted to the adaxial surface of the nectary, yet the annular nectary parenchyma might permit enough nectar secretion to encourage hawkmoth visitation. Raju and Rani (2016) reported an average nectar volume of 0.26 ± 0.10 μL for *G. gynandra* and noted *A. viscosa* produces a trace amount of nectar, insufficient for nectar volume quantification [18]. This finding is consistent with the hypothesis that the volume of nectary parenchyma is correlated to nectar production, as *A. viscosa* has a smaller amount of nectary parenchyma compared with *G. gynandra*. Though both *G. gynandra* and *P. dodecandra* have more extensive nectary parenchyma than *A. viscosa*, Higuera-Díaz et al. (2015) measured a higher average volume of nectar for the generalist species *P. dodecandra* (0.63 ± 0.32 μL) [17], with lower energy requirement pollinators including bees, wasps, and flies. Additional pollination and nectar studies are required to confirm the relationship between the amount of nectary parenchyma, volume of nectar, and pollinator type.

*Cleome violacea* differs from the other eight species in that it does not have prominent nectary parenchyma at anthesis. However, the nectary tends to appear more red-stained earlier in development. Commonly, photosynthate is transported from elsewhere in the plant and stored as starch in the nectary parenchyma [4]. Starch accumulation occurs in the nectary parenchyma of *Arabidopsis thaliana* (Brassicaceae; the sister family to Cleomaceae) [46]. The degradation of starch acts a carbohydrate source for nectar, allowing for nectar production at any time of the day [4]. In ornamental tobacco (Solanaceae), starch accumulates in the nectary parenchyma during development but is rapidly broken down one day before anthesis [46]. Perhaps, the nectary of *Cleome violacea* may be more densely stained earlier in development due to the presence of starch which is subsequently broken down prior to anthesis in preparation for nectar secretion. Carey et al. (2023) reported a low average nectar volume (0.17 ± 0.07 μL) for *Cleome violacea* that decreased with daily collection [36]. The small volume of secreted nectar does not cover the adaxial surface of the nectary lobes (Figure 4A). Like glistening nectar, the glossy exposed surface of the nectary may act as a cue for pollinators by reflecting incident light [47]. Additionally, as the nectary of *Cleome violacea* is a prominent component of the flower, its nectary size may play a role in pollinator attraction.

### 3.3. Evolutionary Lability in Floral Nectary Morphology across Cleomaceae

**Figure 12:**
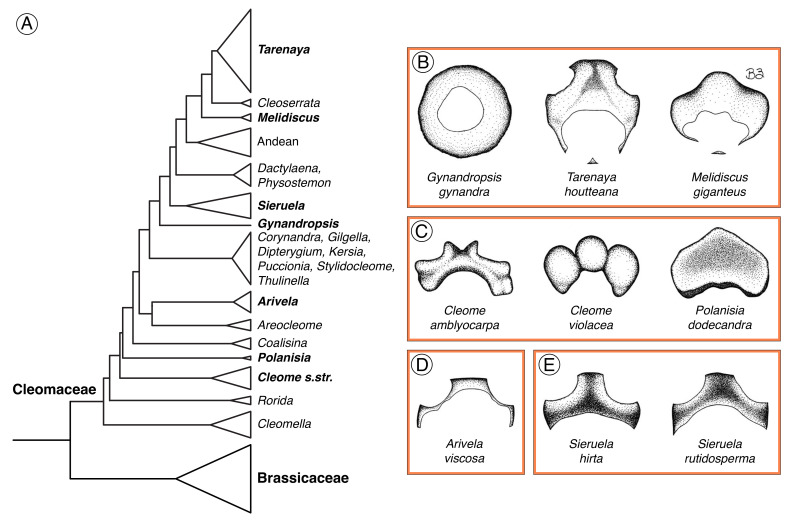
Cleomaceae phylogeny and floral nectaries of the nine species grouped by shape and position. (**A**) Cleomaceae phylogenetic tree derived from that of Patchell et al. (2014) and Bayat et al. (2018) [7,22] with clades sampled here bolded. (**B**) Annular nectaries. (**C**) Protruding adaxial nectaries. (**D**) Slightly convex adaxial nectary. (**E**) Concave adaxial nectaries. Illustrations depict the apical view of the nectary with stippling to represent more basal or concave regions. Nectary size varies between species; illustrations are not to the same scale.

While unified by nectarostomata and vasculature, the diversity in floral nectary location, size, and shape has no clear evolutionary pattern across the family (Figure 12). The floral nectaries of the focal Cleomaceae species can be categorized by shape and position as follows: annular (*G. gynandra*, *T. houtteana*, *M. giganteus*; Figure 12B), protruding adaxial (*Cleome amblyocarpa*, *Cleome violacea*, *P. dodecandra*; Figure 12C), slightly convex adaxial (*A. viscosa*; Figure 12D), and concave adaxial (*S. hirta*, *S. rutidosperma*; Figure 12E). The annular and protruding adaxial nectaries are not confined to a specific clade or genus. Previous research fills in the gaps for some of the genera without representative species in our study; *Cleomella* species have an annular nectary protruding off the receptacle between the perianth and stamens [12,17,24] and *Rorida* species have petal appendages that act as nectaries [15]. Thus, Cleomaceae floral nectaries are not exclusively receptacular, but can also be derived from other organs. In addition, the annular nectaries of *Cleomella*, *Gynandropsis*, *Melidiscus*, and *Tarenaya* are scattered across the phylogeny and Brassicaceae nectaries range from annular to two, four, or eight discrete sections [4]. Although Iltis (1958) hypothesized that adaxial nectaries are derived from annular nectaries, this distribution in nectary shape and position does not clearly support that evolutionary pathway [13]. That is, annular nectary parenchyma could be a derived character state associated with a shift to high energy requirement pollinators. Further, nectar secretion tends to occur exclusively on the adaxial side of the flower regardless of nectary parenchyma positioning. Adaxial nectar secretion could be selected for so that pollinators have easy access to nectar and are likely to contact the upward curving reproductive organs.

In addition, the degree of floral nectary similarity within genera can vary drastically. The nectaries of *S. hirta* and *S. rutidosperma* are similar in shape with a concavity extending from the lateral nectary lobes between the adaxial petals and basally fused adaxial filaments, to the medial nectary lobe between the adaxial petals. The primary difference being the angle of the basally fused adaxial filaments which function to hold the nectar in place. The wall of fused filaments is linear in *S. hirta* and curved toward the adaxial sepal in *S. rutidosperma*. Lunau et al. (2020) briefly describe *Sieruela monophylla* as having a glossy annular false floral nectary [47]. As the flowers of all nine Cleomaceae species described here have nectar-secreting structures and species within the same genera (*S. hirta* and *S. rutidosperma*) have adaxial nectaries, verification of *S. monophylla*’s annular false nectary is needed. Although *Cleome amblyocarpa* and *Cleome violacea* both have nectaries that protrude off the receptacle, the shape of the nectaries is entirely different. *Cleome amblyocarpa* has a somewhat pelvis-shaped nectary, while *Cleome violacea* has a three-lobed nectary. Similarly, Iltis (1958) described considerable within-genera differences in nectary size and shape for *Polanisia*, with nectaries ranging from solid with a concave or truncate apex to tubular [13]. Hence, floral nectary structure is diverse across Cleomaceae, and the drastic variation can also extend to within genera.

## 4. Materials and Methods

### 4.1. Plant Material

Nine species from the Cleomaceae family were sampled: *Arivela viscosa* accession 815 from Hortus Botanicus; *Cleome amblyocarpa* accession 151485 from Royal Botanic Gardens Kew; *Cleome violacea* accession 813 from Hortus Botanicus; *Gynandropsis gynandra* accession TOT8917 kindly provided by M. Eric Schranz, Wageningen University; *Melidiscus giganteus* accession 814 from Hortus Botanicus; *Polanisia dodecandra* accession 68456 from B & T World Seeds; *Sieruela hirta* accession 74520 from B & T World Seeds; *Sieruela rutidosperma* accession 512496 from B & T World Seeds; and *Tarenaya houtteana* accession FL2400 from West Coast Seeds. Seeds were grown in professional growing mix (Sun Gro Horticulture, Agawam, Massachusetts, USA) in University of Alberta Department of Biological Sciences growth chambers set to 28 °C day/22 °C night temperatures with a 12 h day/12 h night cycle. Voucher specimens were deposited at the University of Alberta Vascular Plant Herbarium (ALTA) (see Table S1 for ALTA accession numbers). Fresh flowers were photographed using a Pixel 5 (Google, Menlo Park, CA, USA) alone or attached to a SMZ1500 stereo microscope (Nikon, Tokyo, Japan) with a NexYZ 3-axis universal smartphone adaptor (Celestron, Torrance, CA, USA).

### 4.2. Scanning Electron Microscopy

For each species, flowers were binned into three developmental stages, (1) bud stage, (2) intermediate stage, and (3) anthetic stage (see Table S2 for stage descriptions). Flowers at the three developmental stages were fixed in FAA (50% ethanol, 10% formalin, and 5% glacial acetic acid) on ice under vacuum for 30 min and stored at 4 °C. Fixed specimens were dehydrated in an ethanol series and critical point dried with carbon dioxide using a CPD 030 critical point dryer (Bal-Tec AG, Liechtenstein, Germany). Dried specimens were dissected and mounted on scanning electron microscopy stubs with conductive carbon tabs, sputter coated with gold using a Hummer 6.2 sputter coater (Anatech USA, Sparks, NV, USA) and imaged using a ZEISS EVO 10 scanning electron microscope or a ZEISS Sigma 300 VP field emission scanning electron microscope (Carl Zeiss AG, Oberkochen, Germany). Contrast and brightness of the micrographs were adjusted using Adobe Photoshop but no other modifications were made.

### 4.3. Histological Preparations

Flowers from the nine species at the three developmental stages were fixed in FAA and dehydrated in an ethanol series as previously mentioned. Samples were then cleared with CitriSolv (Decon Labs, King of Prussia, PA, USA), embedded in Paraplast Plus (Sigma-Aldrich, St. Louis, MI, USA), and stored at 4 °C. Samples were sectioned to 8 μm using a Microm HM 325 rotary microtome (Thermo Scientific, Waltham, MA, USA) and mounted on glass slides. Transverse and longitudinal sections were prepared for each species.

### 4.4. Fast Green and Safranin O Staining

Johansen’s fast green and safranin O protocol (1940) as adapted by Ruzin (1999) yields vibrantly stained plant tissues, yet it utilizes several hazardous chemicals [48,49]. Because methyl cellosolve, xylene, and picric acid are toxic to humans and picric acid is highly explosive when dry and can react to form explosive substances [50,51,52], we substituted these chemicals with less harmful alternatives. Historically, ethanol was used as a dehydrating agent but was replaced with methyl cellosolve, before the harmful properties of methyl cellosolve were known [53]. Therefore, we reverted to anhydrous ethanol and used CitriSolv and hydrochloric acid in place of xylene and picric acid, respectfully. The detailed modified protocol is as follows.

Sectioned specimens were deparaffinized and rehydrated by placing slides in the following solutions: CitriSolv for 10 min, fresh CitriSolv for 10 min, 50% CitriSolv and 50% ethanol for 10 min, 100% ethanol for 5 min, 95% ethanol for 5 min, and 70% ethanol for 5 min. Slides were left overnight, approximately 16 h, in safranin O staining solution (1% *w*/*v* safranin O, 1% (*w*/*v*) sodium acetate, 2% (*v*/*v*) formalin, 3 volumes 100% ethanol, and 1 volume deionized water). Excess safranin O staining solution was washed away by submerging slides in deionized water then gently rinsing with deionized water in a squeezable wash bottle. To differentiate safranin O and dehydrate sectioned specimens, slides were placed in the following solutions: 95% ethanol and 0.5% hydrochloric acid for 10 s, 95% ethanol with 4 drops of ammonium hydroxide per 100 mL for 10 s, and 100% ethanol for 10 s. Sectioned specimens were counterstained in fast green staining solution (0.075% (*w*/*v*) fast green FCF, 2 volumes of 100% ethanol, and 1 volume methyl salicylate) for 10 s. To clear sectioned specimens, slides were placed in clearing solution (2 volumes methyl salicylate, 1 volume 100% ethanol, and 1 volume CitriSolv) for 10 s, CitriSolv with 3 drops of 100% ethanol for 3 s, CitriSolv for 5 s, and left in fresh CitriSolv until coverslips were mounted with Permount (Fisher Scientific, Waltham, MA, USA) to avoid drying out the sectioned specimens. Slides were imaged using a Pixel 5 attached to an Eclipse 80i light microscope (Nikon, Tokyo, Japan) with a NexYZ 3-axis universal smartphone adapter. Backgrounds were removed from photographs using the ‘Magic Eraser Tool’ in Adobe Photoshop. The terms adaxial and abaxial refer to the position relative to the floral axis (unless otherwise noted), thus indicating the top and bottom halves of the flower, respectively. The terms frontal and sagittal are used to indicate the type of longitudinal section: (1) a frontal section through the middle of a Cleomaceae flower bisects the lateral sepals and (2) a sagittal section bisects the adaxial and abaxial sepals.

## 5. Conclusions

Though floral nectaries secrete a crucial reward for pollinators, description of nectary structure and development across and within families is lagging [4]. As with other characteristics associated with pollinator interactions such as petal colour and patterns and floral organ number and elaboration, floral nectaries are a morphologically diverse feature across Cleomaceae. As such, detailed descriptions of floral nectaries would be a valuable addition to floras for the identification of Cleomaceae species. Although Cleomaceae floral nectaries vary in colour, size, and shape, they are most commonly receptacular features with nectary parenchyma often extending from the apex of the nectary to the level of perianth attachment. This variation is ideal for exploring outstanding questions regarding the genetic controls of floral nectary size, shape, and parenchyma position. With muddled boundaries of the nectary and receptacle, the receptacle does not always appear to be a well-defined floral organ. Although modifications of the receptacle such as nectaries and the androgynophore contribute to floral diversity in Cleomaceae [54] and receptacular nectaries are common across flowering plants [4], the receptacle is often overlooked in floral evo-devo studies. Thus, the involvement of the receptacle in floral diversification and pollinator interactions necessitates further investigation.

## Data Availability

The data presented in this study are included in this article and Supplemental Material.

## Supplementary Materials

The following supporting information can be downloaded at: https://www.mdpi.com/article/10.3390/plants12061263/s1, Table S1: Accession numbers for the nine Cleomaceae species voucher specimens; Table S2: Brief descriptions of the flowers at the three developmental stages for the nine Cleomaceae species.
