# Supplementary material for: Comparative Nectary Morphology across Cleomaceae (Brassicales)
Source: Plants (Basel). 2023 Mar 10;12(6):1263. doi: 10.3390/plants12061263 (PMC10051628; doi:10.3390/plants12061263)
Supplement: Supplementary file 1 [file plants-12-01263-s001.zip › Table_S1.pdf]

Table S1. Accession numbers for the nine Cleomaceae species voucher specimens. All voucher specimens were collected by B.Z. or B.Z. and S.W. and deposited in the University of Alberta Vascular Plant Herbarium (ALTA).

Species	ALTA Accession Number
Arivela viscosa	143371
Cleome amblyocarpa	144822
Cleome violacea	144828
Gynandropsis gynandra	143369
Melidiscus giganteus	144833
Polanisia dodecandra	144836
Sieruela hirta	143370
Sieruela rutidosperma	144838
Tarenaya houtteana	144840
