# Supplementary material for: Comparative Nectary Morphology across Cleomaceae (Brassicales)
Source: Plants (Basel). 2023 Mar 10;12(6):1263. doi: 10.3390/plants12061263 (PMC10051628; doi:10.3390/plants12061263)
Supplement: Supplementary file 1 [file plants-12-01263-s001.zip › Table_S2.pdf]

Table S2. Brief descriptions of the flowers at the three developmental stages for the nine Cleomaceae species.

Species	Stage	Description
Arivela viscosa	Bud	Sepals closed with petals inserted; 0.4–0.6 cm from base of sepals to apex of sepals
	Intermediate	Sepals closed with petals exserted; 0.7–0.9 cm from base of sepals to apex of petals
	Anthesis	Anthesis; sepals and petals open, anthers dehisced
Cleome amblyocarpa	Bud	Sepals closed with petals inserted; 0.2 cm from base of sepals to apex of sepals
	Intermediate	Sepals closed with petals exserted; 0.5–0.6 cm from base of sepals to apex of petals
	Anthesis	Anthesis; sepals and petals open, anthers dehisced
Cleome violacea	Bud	Sepals closed with petals exserted; 0.1–0.4 cm in length from base of sepals to apex of petals
	Intermediate	Petals beginning to open
	Anthesis	Anthesis; sepals and petals open, anthers dehisced
Gynandropsis gynandra	Bud	Sepals closed with petals inserted, apex of pistil level with sepals or exserted; 0.5–0.7 cm from base of sepals to apex of pistil
	Intermediate	Sepals closed with petals exserted, pistil elevated above petals; 1.5–1.9 cm from base of sepals to apex of pistil
	Anthesis	Anthesis; sepals and petals open, anthers dehisced
Melidiscus giganteus	Bud	Sepals closed with abaxial sepal apex extending beyond closed petals; 1.5–2.5 cm from base of sepals to apex of petals
	Intermediate	Sepals open, filaments and gynophore exserted and incurved with anthers and pistil enclosed in petals
	Anthesis	Anthesis; sepals and petals open, anthers and pistil released from petals
Polanisia dodecandra	Bud	Sepals closed with petals inserted, beak of pistil exserted; 0.5–0.7 cm from base of sepals to apex of pistil
	Intermediate	Sepals and petals opening, filaments not fully elongated; 1.0–1.2 cm from base of sepals to apex of pistil
	Anthesis	Anthesis; sepals and petals open, anthers dehisced
Sieruela hirta	Bud	Sepals closed with petals inserted; 0.4–0.9 cm from base of sepals to apex of sepals
	Intermediate	Sepals closed with petals exserted; 1.0–1.5 cm from base of sepals to apex of petals
	Flower	Anthesis; sepals and petals open, anthers dehisced
Sieruela rutidosperma	Bud	Sepals closed with petals exserted; 0.3–0.5 cm from base of sepals to apex of petals
	Intermediate	Sepals closed with petals exserted; 0.6–0.7 cm from base of sepals to apex of petals
	Anthesis	Anthesis; sepals and petals open, anthers dehisced
Tarenaya houtteana	Bud	Sepals closed with petals exserted; 0.7–1.9 cm from base of sepals to apex of petals
	Intermediate	Sepals open, filaments and gynophore exserted and incurved with anthers and pistil enclosed in petals
	Anthesis	Anthesis; sepals and petals open, anthers and pistil released from petals

Note: Descriptions include key distinguishing features for each stage. As the floral nectaries are obscured early in development for all species and at anthesis for some species, nectary characteristics were excluded from the descriptions.
